# Higher Right Hemisphere Gamma Band Lateralization and Suggestion of a Sensitive Period for Vocal Auditory Emotional Stimuli Recognition in Unilateral Cochlear Implant Children: An EEG Study

**DOI:** 10.3389/fnins.2021.608156

**Published:** 2021-03-09

**Authors:** Giulia Cartocci, Andrea Giorgi, Bianca M. S. Inguscio, Alessandro Scorpecci, Sara Giannantonio, Antonietta De Lucia, Sabina Garofalo, Rosa Grassia, Carlo Antonio Leone, Patrizia Longo, Francesco Freni, Paolo Malerba, Fabio Babiloni

**Affiliations:** ^1^Laboratory of Industrial Neuroscience, Department of Molecular Medicine, Sapienza University of Rome, Rome, Italy; ^2^BrainSigns Srl, Rome, Italy; ^3^Cochlear Implant Unit, Department of Sensory Organs, Sapienza University of Rome, Rome, Italy; ^4^Audiology and Otosurgery Unit, “Bambino Gesù” Pediatric Hospital and Research Institute, Rome, Italy; ^5^Otology and Cochlear Implant Unit, Regional Referral Centre Children’s Hospital “Santobono-Pausilipon”, Naples, Italy; ^6^Department of Otolaryngology/Head and Neck Surgery, Monaldi Hospital, Naples, Italy; ^7^Department of Otorhinolaryngology, University of Messina, Messina, Italy; ^8^Cochlear Italia Srl, Bologna, Italy; ^9^Department of Computer Science and Technology, Hangzhou Dianzi University, Xiasha Higher Education Zone, Hangzhou, China

**Keywords:** lateralization index, right hemisphere emotion hypothesis, deafness, hearing loss, brain activity, length of cochlear implant use, sensitive period, auditory age

## Abstract

In deaf children, huge emphasis was given to language; however, emotional cues decoding and production appear of pivotal importance for communication capabilities. Concerning neurophysiological correlates of emotional processing, the gamma band activity appears a useful tool adopted for emotion classification and related to the conscious elaboration of emotions. Starting from these considerations, the following items have been investigated: (i) whether emotional auditory stimuli processing differs between normal-hearing (NH) children and children using a cochlear implant (CI), given the non-physiological development of the auditory system in the latter group; (ii) whether the age at CI surgery influences emotion recognition capabilities; and (iii) in light of the right hemisphere hypothesis for emotional processing, whether the CI side influences the processing of emotional cues in unilateral CI (UCI) children. To answer these matters, 9 UCI (9.47 ± 2.33 years old) and 10 NH (10.95 ± 2.11 years old) children were asked to recognize nonverbal vocalizations belonging to three emotional states: positive (achievement, amusement, contentment, relief), negative (anger, disgust, fear, sadness), and neutral (neutral, surprise). Results showed better performances in NH than UCI children in emotional states recognition. The UCI group showed increased gamma activity lateralization index (LI) (relative higher right hemisphere activity) in comparison to the NH group in response to emotional auditory cues. Moreover, LI gamma values were negatively correlated with the percentage of correct responses in emotion recognition. Such observations could be explained by a deficit in UCI children in engaging the left hemisphere for more demanding emotional task, or alternatively by a higher conscious elaboration in UCI than NH children. Additionally, for the UCI group, there was no difference between the CI side and the contralateral side in gamma activity, but a higher gamma activity in the right in comparison to the left hemisphere was found. Therefore, the CI side did not appear to influence the physiologic hemispheric lateralization of emotional processing. Finally, a negative correlation was shown between the age at the CI surgery and the percentage of correct responses in emotion recognition and then suggesting the occurrence of a sensitive period for CI surgery for best emotion recognition skills development.

## Introduction

Processing emotional expressions is fundamental for social interactions and communication; in fact, from a very young age, infants are able to detect visual and auditory information in faces and voices of people around them (Grossmann, 2010). Such capability would develop into the skill to recognize and discriminate emotions, thanks to the contribution of the experience and of the maturation of sensory and perceptual systems. This recognition involves a multisensory effect, evidenced by integration effects of facial and vocal information on cerebral activity, which are apparent both at the level of heteromodal cortical regions of convergence (e.g., bilateral posterior superior temporal sulcus), and at unimodal levels of sensory processing (Campanella and Belin, 2007; Davies-Thompson et al., 2019; Young et al., 2020).

In relation to such cross-sensorial and unisensorial effects, hearing impairment could compromise multisensory integration, in relation to its onset, etiology, and severity, leading the patient to rely only or predominantly on the visual modality in communication, including emotional perception and expression (Mildner and Koska, 2014). In fact, for 92% of children with cochlear implant (CI), perception was dominated by vision when visual and auditory speech information conflicted (Schorr et al., 2005). This statement is supported by the results of studies employing the McGurk effect on CI users, which requires the integration of auditory and visual sensory stimuli. For instance, children who received their CI prior to age 30 months accurately identified the incongruent auditory–visual stimuli, whereas children who received their CI after 30 months of age did not (Schorr, 2005). This evidence appears particularly worthy because differently from adults, who mainly prefer visual modality, infants and young children show auditory processing preference, but in children with congenital hearing impairment, such auditory dominance appears absent. Interestingly, in post-lingually deaf CI patients, such greater relying on visual information, indexed by higher speech-reading performances than normal-hearing (NH) individuals, led instead to an increased capacity of integrating visual and distorted speech signals, producing higher visuoauditory performances (Rouger et al., 2007). Furthermore, such evidence in post-lingual deaf patients was also supported by neurophysiological assessments, evidencing a positive correlation between visual activity and auditory speech recovery, suggesting a facilitating role for the visual modality in auditory words’ perception during communicative situations (Strelnikov et al., 2013). With respect to general processing preferences, contrary to adults, who prefer the visual modality (Scherer, 2003), infants and young children exhibit auditory processing preference. Importantly, congenital hearing-impaired children who underwent auditory–verbal therapy (a therapy limiting visual cue in order to strengthen the auditory pathway for language learning) reported a behavior similar to NH children, which is an overall auditory preference in response to audiovisual stimuli, although responses did not significantly differ from chance (Zupan and Sussman, 2009). Contrary to NH individuals, those with hearing impairments do not benefit from the addition of the auditory cues to the visual mode (e.g., Most and Aviner, 2009). Although the accuracy of emotion perception among children with hearing loss (HL) was lower than that of NH children in auditory, visual, and auditory–visual conditions, in prelingually deaf very young children (about 4–6 years old), the combined auditory–visual mode significantly surpassed the auditory or visual modes alone, as in the NH group, supporting the use of auditory information for emotion perception, probably thanks to intensive rehabilitation (Most and Michaelis, 2012) and neuroplasticity. Such results strongly support the hypothesis of a sensitive period (Kral et al., 2001; Sharma et al., 2005; Gilley et al., 2010) for the establishment of the integration of auditory and visual stimuli.

Thanks to their activity of direct stimulation of the acoustic nerve, converting the auditory stimuli into electrical signals directed to the brain, CIs can successfully restore hearing in profoundly deaf individuals. After intensive rehabilitation, most CI users can reach a good level of speech comprehension. However, the acoustic signal provided by the device is severely degraded, resulting in a poor frequency resolution and deficits in pitch patterns (Gfeller et al., 2007; eHopyan et al., 2012) and pitch changes or direction discrimination (Gfeller et al., 2002) in comparison to NH controls.

Hearing-impaired children go through an early auditory development that is different from that of NH toddlers. This condition would affect their judgment of the emotional content of a stimulus, insofar as the auditory modality resulted as particularly important for the communication of emotions in young children (Baldwin and Moses, 1996; Akhtar and Gernsbacher, 2008). The study of such mechanisms appears of great impact since about 600,000 patients world-wide are CI users (The Ear Foundation, 2017), and many of them are children who were born deaf or lost their hearing within the first few years of life. CI children are a paradigmatic model for the study of emotion recognition skills, as due to the early acquisition of deafness, they learned language through the degraded input of the CI, which greatly affects harmonic pitch perception. This ability is strongly necessary for emotion recognition in voices, and its deficiency could have implications on how child CI users learn to produce vocal emotions (Damm et al., 2019). However, a very recent study provided evidence that also deaf people can develop skills for emotional vocalizations despite the presence of some differences in comparison to NH adults (Sauter et al., 2019). Using unilateral CI (UCI) in children, due to non-physiological development of their auditory system and to their asymmetry in receiving auditory inputs, represents a powerful model of investigation of the possible modulation of the hemispheric specialization and of auditory-related emotional skills development in relation to the restored hearing condition. Additionally, such participants would provide evidence of the possible modulation of the physiological processes of emotion recognition following the restoration of the auditory capabilities, of which the exact time of beginning is due to the CI surgery time. Children, 7–13 years of age, using UCIs perform more poorly than age- and gender-matched controls on the affective speech prosody task but as well as controls in tasks of facial affect perception (Hopyan-Misakyan et al., 2009), as measured by the DANVA-2 (Nowicki and Duke, 1994).

One of the few studies that investigated both auditory recognition and vocal production of emotions did not find any consistent advantage for age-matched NH participants in comparison to three prelingually, bilaterally, profoundly deaf children aged 6–7 years who received CIs before age 2 years; however, confusion matrices among three of the investigated emotions (anger, happiness, and fear) showed that children with and without hearing impairment may rely on different cues (Mildner and Koska, 2014).

With respect to emotional skills attainment and in relation to the hemispheric specialization for emotional processing (Gainotti, 2019), it is interesting to consider that patients enrolled in the present study were UCI users, that is, single-side deaf (SSD) patients. In fact, in SSD population, it was evidenced that the occurrence of a massive reorganization of aural preference in favor of the hearing ear is greater than the precocity of unilateral HL onset, therefore supporting the importance of a short time between the first and second implantation in children (Kral et al., 2013; Gordon et al., 2015; Gordon and Papsin, 2019).

Concerning neural correlates of emotion recognition, gamma band electroencephalogram (EEG) was found to be particularly sensitive for emotion classification (Li and Lu, 2009; Yang et al., 2020). Gamma band cerebral activity has been previously linked to facial emotion recognition processes; for instance, a right hemisphere dominance in gamma activity was found during emotional processing of faces in comparison to neutral ones (e.g., Balconi and Lucchiari, 2008). Such evidences are in accord to the right hemisphere hypothesis for emotion processing, that starting from observations on patients with single hemisphere lesions states the dominance of the right hemisphere for every kind of emotional response (Gainotti, 2019). With specific regard to emotional prosody processing and brain activity lateralization, Kotz and colleagues hypothesized that (i) differentially lateralized subprocesses underlie emotional prosody processing and (ii) the lateralization of emotional prosody can be modulated by methodological factors (Kotz et al., 2006). Furthermore, concerning verbal stimuli, in adult CI users, gamma band–induced activity was found to be higher in NH than in CI users, irrespectively of the valence of the emotions investigated (Agrawal et al., 2013).

On the base of the previous issues, the following experimental questions have been approached in a population of NH and UCI children: (i) Given the non-physiological development of the auditory system in deaf children who underwent hearing restoration through CI use, are the emotional auditory stimuli processed in a similar way than NH children? (ii) Is the auditory age, meant as the age at CI surgery, crucial in the capacity of recognizing emotions? (iii) In light of the evidence that the right hemisphere has a unique contribution in emotional processing – summarized in the right hemisphere emotion hypothesis – does the side of the CI influence the processing of emotional cues in UCI children, or is the “physiological right lateralization” respected?

## Materials and Methods

### Participants

For the present study, 10 NH (6 female, 4 male; 10.95 ± 2.11 years old) and 9 UCI user (UCI; 5 female, 4 male; 9.47 ± 2.33 years old) children were enrolled. Six children had their CI in their right ear and three in their left ear; at the moment of the test, none of them wore any hearing aid in their contralateral ear. All participants were right-handed except for two children: one belonging to the NH and one to the UCI group. Further clinical details of the UCI group are reported in Table 1.

**TABLE 1:** Demographics concerning the UCI group, in particular etiology of deafness, its onset, and duration of deafness before CI surgery.

Participants	Age (years)	Etiology	Onset of deafness	Period of Deafness (years)
PI	11,39	Unknown	Birth	1,38
P2	12,04	Unknown	3 years old	5,91
P3	11,66	Unknown	4 years old	2,25
P4	10,22	Homozygous mutation of the connexin-26 gene	Birth	1,11
P5	7,08	Congenital CMV infection	Birth	3,82
P6	9,99	Homozygous mutation of the connexin-26 gene	Birth	2,93
P7	9,24	Homozygous mutation of the connexin-26 gene	Birth	8,16
P8	12,57	Unknown	3,5 years old	6,41
P9	14,37	Unknown	Birth	13,18

### Protocol

**FIGURE 1:**
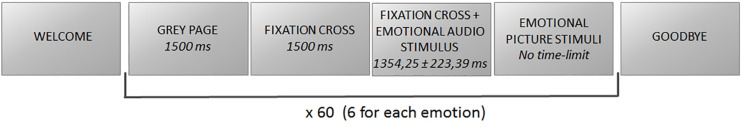
Scheme of the experimental protocol.

The task consisted of the recognition of nonverbal vocalizations belonging to a database previously validated and employed in several studies (Sauter et al., 2006, 2010, 2013) and grouped into three emotional states: positive (achievement, amusement, contentment, relief), negative (anger, disgust, fear, sadness), and neutral (neutral, surprise), which participants were asked to match with the corresponding emotional picture (Figure 1). For each emotion, six different audio stimuli were reproduced, whereas there was a single corresponding emotional picture for each emotion. The emotional audio stimuli had a mean duration of 1,354.25 ± 223.39 ms and were delivered at 65 dB HL (Cartocci et al., 2015, 2018; Marsella et al., 2017; Piccioni et al., 2018) through two loudspeakers placed in front of and behind the participant at the distance of 1 m each, to meet CIs’ best requirements for their use. Participants underwent training with the kind of emotional stimuli employed in the study and a familiarization with the experimental protocol. Once the researcher verified the comprehension of the emotional stimuli and the task by the participant, he/she was asked to carefully listen to the emotional audio and then to identify the emotion reproduced by the stimulus pressing one out of five buttons on a customized keyboard, corresponding to the target emotional picture. For instance, the participant heard a laugh, and he/she had to identify the corresponding picture, a smiling young lady, out of five options. There was no time limit set for such identification and matching with the target emotion. Each picture representing the target emotion was placed at least once (and maximum twice) in each of the five positions on the screen. The number of five pictures among which the participant had to identify the target stimulus was chosen in accordance with Orsini et al. (1987), who found for the range of age of the enrolled participants a digit span of more than 4.5 items for both males and females. Stimuli were delivered through E-prime software, in a pseudorandomized order so that it was not possible that two stimuli belonging to the same emotion were consecutive.

The study was carefully explained to all participants and to their parents, who signed an informed consent to the participation. The study was approved by the Bambino Gesù Pediatric Hospital Ethic Committee, protocol 705/FS, and was conducted according to the principles outlined in the Declaration of Helsinki of 1975, as revised in 2000.

### EEG

A digital EEG system (BE plus EBNeuro, Italy) was used to record 16 EEG channels (Fp, Fz, F3, F4, F7, F8, T7, T8, P3, P4, P7, P8, O1, O2) according to the international 10/20 system, with a sampling frequency of 256 Hz. The impedances were maintained below 10 kΩ, and a 50-Hz notch filter was applied to remove the power interference. A ground electrode was placed on the forehead and reference electrodes on earlobes. The EEG signal was initially bandpass filtered with a fifth-order Butterworth filter (high-pass filter: cutoff frequency fc = 1 Hz; low-pass filter: cutoff frequency fc = 40 Hz). Because we could not apply independent component analysis because of the low number of EEG channels (i.e., 16), we used a regression-based method to identify and correct eye-blinks artifacts. In particular, the Fpz channel was used to identify and remove eye-blink artifacts by the REBLINCA algorithm (Di Flumeri et al., 2016). This method allows the EEG signal to be corrected without losing data. For other sources of artifacts (e.g., environmental noise, user movements, etc.), specific procedures of the EEGLAB toolbox were employed (Delorme and Makeig, 2004). In particular, the EEG dataset was first segmented into epochs of 2 s through moving windows shifted by 0.125 s. This windowing was chosen with the compromise of having a high number of observations, in comparison with the number of variables, and in order to respect the condition of stationarity of the EEG signal. This is in fact a necessary assumption in order to proceed with the spectral analysis of the signal. Successively, three criteria were applied to those EEG epochs (Aricò et al., 2017; Borghini et al., 2017): (i) threshold criterion (amplitudes exceeding ± 100 μV); (ii) trend criterion (slope higher than 10 μV/s); and (iii) sample-to-sample criterion (sample-to-sample amplitude difference >25 μV).

All EEG epochs marked as “artifact” were removed in order to have a clean EEG signal. In order to accurately define EEG bands of interest, for each participant the individual alpha frequency (IAF) was computed on a closed-eyes segment recorded prior to the experimental task. Thus, the EEG was filtered in the following frequency bands: theta [IAF − 6 ÷ IAF − 2 Hz], alpha [IAF − 2 ÷ IAF + 2 Hz], beta [IAF + 2 ÷ IAF + 16 Hz], and gamma [IAF + 16 ÷ IAF + 25 Hz] (Klimesch, 1999). EEG recordings were segmented into trials, corresponding to audio stimulus listening and target picture matching. The power spectrum density was calculated in correspondence of the different conditions with a frequency resolution of 0.5 Hz. Trials were normalized by subtracting the open-eyes activity recorded before the beginning of the experimental task.

### Lateralization Index

The lateralization index (LI) was calculated in order to assess the relative asymmetry between the two cerebral hemispheres’ activity during the task execution (audio stimuli perception and target visual stimuli matching), as the right hemisphere theory for emotion predicts a relative higher right activation during emotional stimuli processing.

The LI was calculated on the basis of the formula previously adopted by Vanvooren et al. (2015):

$$\text{L}\,I=\frac{\text{R}-\text{S}}{\text{RS}}$$

where *R* stands for right hemisphere, and *L* for left hemisphere. The LI ranges from +1, for cortical activity entirely asymmetrical to the right hemisphere, to zero for symmetrical cortical activity, and −1 for cortical activity entirely asymmetrical to the left hemisphere. For the right hemisphere activity calculation, the estimation from the following electrodes was averaged: F4, F8, T8, P4, P8, O2, whereas for the left hemisphere. It was averaged from the following ones: F3, F7, T7, P3, P7, O1. The LI was already employed on hearing-impaired children, in particular, SSD children, finding an asymmetry in cortical activity during the execution of a word in noise recognition task influenced by the direction of the background noise in SSD but not in NH children (Cartocci et al., 2019).

### Statistical Analysis

Both the percentage of correct responses and LI data were compared between the NH and UCI groups through analysis of variance (ANOVA) with two factors: GROUP (2 levels: NH and UCI) and EMOTIONAL STATE (3 levels: positive, negative, and neutral). A simple regression analysis was performed for investigating the relation between (i) the percentage of correct responses and the LI values, (ii) between the percentage of correct responses and the age at the test execution, and (iii) between the percentage of correct responses and the age at CI surgery.

## Results

**FIGURE 2:**
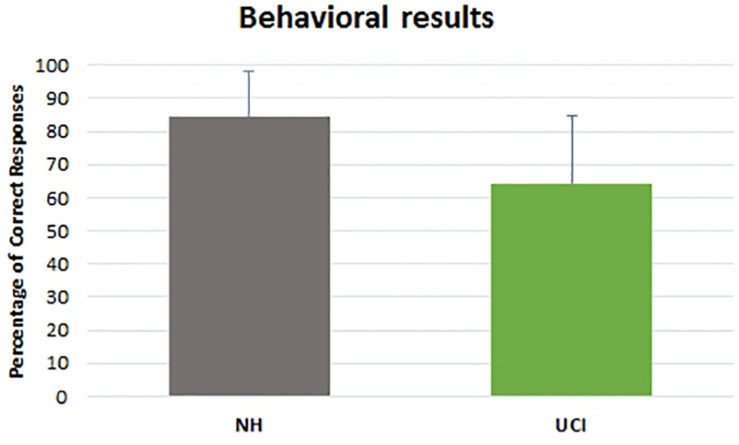
Percentage of correct responses reported by NH and UCI children. Bars describe means, and error bars describe standard deviations.

Behavioral results evidenced a higher percentage of correct responses provided by NH children in comparison to UCI children (*F* = 18.898, *p* < 0.001, partial η^2^ = 0.270) (Table 2), but an effect of the emotional state was not seen (*F* = 1.890, *p* = 0.161, partial η^2^ = 0.069), although for both groups the neutral cues were the most difficult to recognize. Neither the interaction between the variable group and emotional state (*F* = 0.032, *p* = 0.968, partial η^2^ = 0.001) was observed (Figure 2).

**TABLE 2:** Mean percentages of correct responses ± standard deviation for each group (UCI and NH) and for each emotional state.

Group	Negative	Neutral	Positive
NH	86,58% ± 9,82	78,33% ± 18,92	88,33% ± 10,17
UCI	65,05% ± 19,37	58,24% ± 22,17	69,67% ± 21,02

**FIGURE 3:**
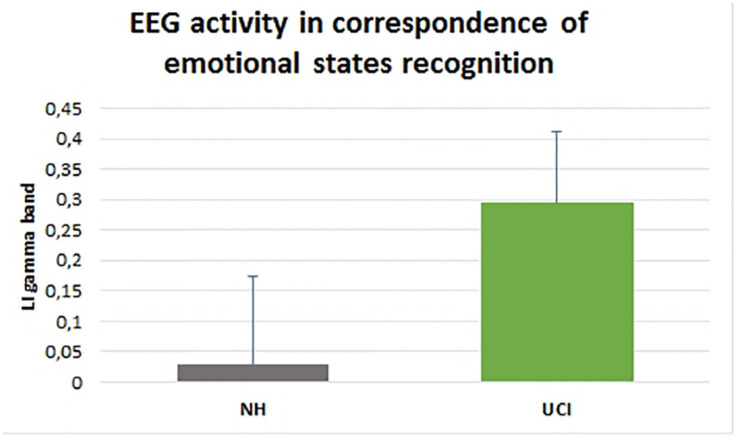
Gamma band activity LI in NH and UCI children. Bars describe means, and error bars describe standard deviations.

ANOVA results showed higher LI values, indicating a higher activity in gamma band in the right in comparison to the left hemisphere, in UCI in comparison to NH children (*F* = 58.656, *p* < 0.001, partial η^2^ = 0.535) (Figure 3), irrespectively of the emotional state (negative, neutral, and positive) (*F* = 1.686, *p* = 0.195, partial η^2^ = 0.062). Additionally, any interaction between the variable groups and emotional state was not found (*F* = 1.121, *p* = 0.333, partial η^2^ = 0.042).

**FIGURE 4:**
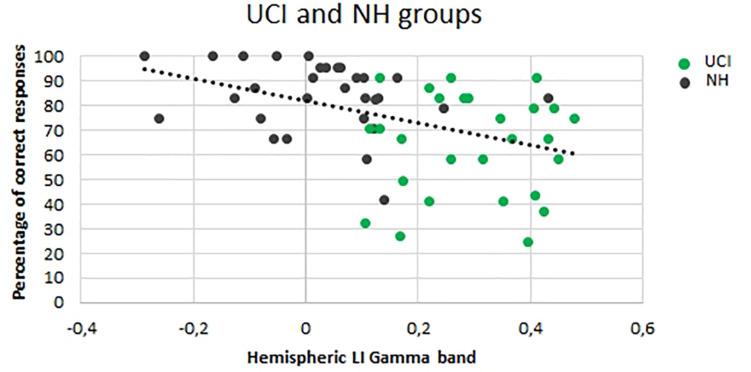
Correlation between the lateralization (LI) gamma values and percentage of correct responses for both the UCI and NH groups. Dark dots represent NH values, and green dots represent UCI values.

A negative correlation was observed between LI gamma values and the percentage of correct responses (*F* = 11.801, *p* = 0.001, *r* = −0.420, partial η^2^ = 0.177) (Figure 4).

**FIGURE 5:**
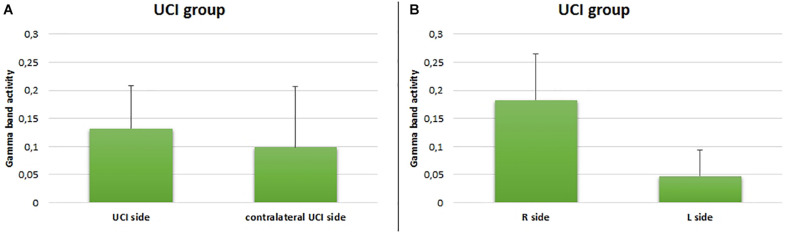
Comparison between gamma activity in the UCI group with respect to the UCI side **(A)** and right or left side **(B)**. Bars describe means, and error bars describe standard deviations.

Additionally, for the UCI group, any difference between the CI side and the deaf contralateral side in the gamma activity was not shown (*F* = 0.598, *p* = 0.212, partial η^2^ = 0.032) (Figure 5A), but a higher gamma activity in the right in comparison to the left hemisphere was found (*F* = 54.552, *p* < 0.001, partial η^2^ = 0.532) (Figure 5B).

**FIGURE 6:**
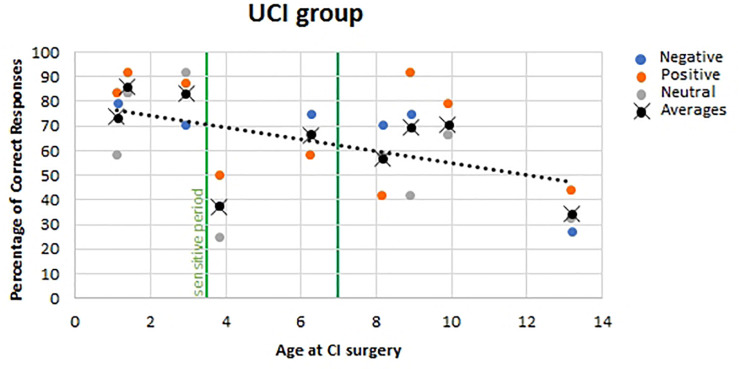
Correlation between age at CI surgery and percentage of correct responses in UCI children. Orange dots stand for positive emotional states; blue dots stand for negative emotional states, and gray dots stand for neutral emotional states. Black dots stand for the mean of correct responses for each participant, irrespective of the emotional state. The vertical green lines represent the sensitive period threshold (3.5 and 7 years old) for the central auditory system development (Sharma et al., 2005).

For the UCI group, no correlation was found between the age of the UCI children at the moment of the experiment and the percentage of correct responses (*F* = 0.052, *p* = 0.821, *r* = 0.046, partial η^2^ = 0.002), similarly to the NH children group (*F* = 1.130, *p* = 0.297, *r* = 0.197, partial η^2^ = 0.039). Additionally, a negative correlation was shown between the age at the CI surgery and the percentage of correct response reported by UCI children (*F* = 7.030, *p* = 0.014, *r* = 0.468, partial η^2^ = 0.219) (Figure 6). Finally, when calculating the mean of the correct responses for each participant, irrespective of the emotional states, despite the lack of significance (*F* = 3.056, *p* = 0.124, *r* = −0.551, partial η^2^ = 0.304), a higher percentage of correct responses was highlighted, higher than 70%, only in early implanted children, that is, before 3.5 years of age (Figure 6, black dots).

## Discussion

According to literature, the lower percentage of correct responses provided by UCI children in comparison to NH children highlights their impairment in vocal emotion recognition skills (Agrawal et al., 2013; Wiefferink et al., 2013; Chatterjee et al., 2015; Jiam et al., 2017; Ahmed et al., 2018; Paquette et al., 2018). This would be strongly related to the preverbal and periverbal deafness acquisition. In fact, in a study employing emotional vocal stimuli in adult CI users, such performance difference was not shown (Deroche et al., 2019). Furthermore, there are evidences of different strategies implemented by CI and NH listeners for emotional stimuli recognition, more based on pitch range cues in the former and more relying on mean pitch in the latter group (Gilbers et al., 2015). In addition, such deficit in emotion recognition in UCI children in comparison to NH children appears strictly related to the matter of social interaction and social development (Jiam et al., 2017); in fact, a correlation between impairments in perception and production of voice emotion was found, like in the case of infant-directed speech, and in 5- to 13-year-old children who used CI (Nakata et al., 2012). It is interesting to note that a previous study employing vocal child-directed happy and sad speech stimuli reported higher performance in NH in comparison to CI using children; however, the percentage of recognition was higher than the one reported in the present study, probably due to the child-directed characteristic of the stimuli (Volkova et al., 2013).

Concerning the difference in gamma LI values observed in UCI in comparison to the NH group, it confirmed a difference in gamma band activity previously reported by Agrawal et al. (2013) in comparison between the same groups, therefore supporting the suitability of the study of gamma rhythms in the investigation of emotional messages conveyed by means of auditory stimuli. However, the previously mentioned study and the present study are not perfectly comparable because of the differences (i) in the sample – adults and children, respectively, – and therefore plausibly in the etiology of deafness; (ii) in the location of EEG activity acquisition, that is, Cz and multiple electrodes over the two hemispheres, respectively; and (iii) in the kind of emotional stimuli, that is, verbal stimuli pronounced with neutral, happy, and angry prosody in Agrawal and colleagues’ study, while vocal nonverbal stimuli belonging to 10 emotions grouped into three emotional states in the present study. Moreover, the higher LI values reported for UCI in comparison to NH children would imply a more sustained conscious processing of the stimuli for the NH group in comparison to the UCI group and a higher processing of the emotional face stimuli – employed for the matching of the auditory stimuli for the identification of the target emotion – by the UCI group (Balconi and Lucchiari, 2008). In fact, McGurk studies showed a higher relying of UCI children on the visual sensation than on the auditory one in case of uncertainty (Schorr et al., 2005).

The correlation between higher right lateralization, as indexed by higher LI values, and the percentage of correct responses could be explained by the evidence of higher activation and asymmetry levels in poorer performers in emotion-in-voice recognition tasks than those of more proficient ones (Kislova and Rusalova, 2009). This possibly also reflects the poorer performance in emotion recognition obtained by UCI children, as well as their higher LI values in comparison to NH children. In fact, it was shown by studies on single hemisphere damage that although the right hemisphere is responsible for low-level discrimination and recognition of affective prosody, in case of higher task demands in terms of associational-cognitive requirements, the left hemisphere is engaged (Tompkins and Flowers, 1985). Thus, UCI children would present deficits in such engaging of the left hemisphere for more complex emotional processing tasks. This could be explained by the neuroimaging evidence that indeed areas appearing to be primarily involved in emotional prosodic processing, that is, posterior temporal (parietal) brain regions (Kotz et al., 2006), are the same areas presumably more involved by the neuroplastic changes that occurred after CI surgery (Giraud et al., 2000; Kang et al., 2004) and the following hearing sensation restoration.

The negative correlation between age of implantation and percentage of correct responses in emotion recognition is in accordance with previous studies (Mancini et al., 2016). On the contrary, in the Deroche and colleagues’ study on adult CI users cited above, any effect of the age at implantation on the emotion recognition was not found, but this would be caused by the post-lingual acquisition of deafness in the majority of the sample (19 over 22 CI users) and by the type of emotions investigated, which is happy, sad, and neutral, whereas in the present study, 10 emotions were employed (Deroche et al., 2019). Furthermore, in Volkova et al.’ (2013) study, employing child-directed emotional speech, performance of the children CI users was positively associated with duration of implant use. Such evidence could be compared to present results, given the almost overlap between age at CI surgery and length of CI use in the enrolled sample. In addition, the trend that better performances were obtained by children implanted before 3.5 years old suggests the influence of a sensitive period, identified through P1 cortical auditory-evoked potential trajectory post-CI development (Sharma et al., 2002, 2005; Sharma and Dorman, 2006; Kral and Sharma, 2012; Kral et al., 2019) also on emotion recognition skills development. Such phenomenon could be explained by the better auditory–visual integration achieved by children implanted before 3.5 years of age as shown by Miller’s test of the race model inequality executed by early and late implanted children (Gilley et al., 2010). Such auditory–visual integration capability achievement is also witnessed by McGurk effect tests on CI children, showing that 38% of early implanted children – before the age of 2.5 years – but none of the late implanted children exhibited the bimodal fusion occurring in the McGurk effect, being instead biased toward the visual modality in contrast to the NH children who were biased toward the audio modality (Schorr et al., 2005). These evidences, with respect to the topic of emotion recognition skills development, are in accord to studies indicating that auditory and visual integration is necessary for the achievement of such capabilities (Campanella and Belin, 2007). In relation to this matter, there is also the evidence of a delay on facial emotion recognition in preschoolers using CI (and hearing aids) in comparison to NH mates, and interestingly, there was not any correlation between facial emotion recognition and language abilities (Wang et al., 2011). Differently, another study found a relation between better language skills and higher social competence, both in NH and CI children, although in the latter group, less adequate emotion-regulation strategies and less social competence than NH children were highlighted (Wiefferink et al., 2012). In addition, a study investigating both linguistic (recognition of monosyllabic words and of key words from sentences within background noise; repetition of non-words) and indexical (discrimination of across-gender and within-gender talkers; identification of emotional content from spoken sentences) properties in perceptual analysis of speech in CI children found an association between better performances in such feature recognition and a younger age at implantation (and use of more novel speech processor technology) (Geers et al., 2013).

Moreover, concerning the emotional communication, a suggestion of deficits also in the imitation of emotional (happy and sad) speech stimuli was found (Wang et al., 2013). Therefore, it sharply results in the vision and need of two targets of rehabilitation for children with CI that should be treated both conjointly and separately: language treatment and emotional intervention.

## Conclusion

In light of the present results, in relation to the experimental questions previously declared, it is possible to conclude that (i) the processing of the emotional stimuli by deaf children using CI appears to be different from NH children, as suggested by the higher relative right hemisphere gamma band activity, possibly explained by the non-physiological development of the auditory system; (ii) on account of the inverse correlation between the age at the CI surgery and the percentage of correct responses, the precocity of performing the CI surgery for the attainment of best emotion recognition skills appears crucial, probably because of neuroplastic changes allowing a better processing and categorization of emotional stimuli; and (iii) the CI side does not appear to influence the processing of emotional stimuli, although interestingly the relative higher gamma band activity appears to be counterproductive in terms of emotion recognition performances; such aspect needs further investigation at the light of the possible particular implications of the right hemisphere hypothesis (Kotz et al., 2006).

## Data Availability Statement

The raw data supporting the conclusions of this article will be made available by the authors, without undue reservation.

## Ethics Statement

The studies involving human participants were reviewed and approved by the Bambino Gesù Pediatric Hospital Ethics Committee. Written informed consent to participate in this study was provided by the participants’ legal guardian/next of kin.

## Author Contributions

GC conceived and conducted the study, performed the data analysis, and wrote the manuscript. AG and BI prepared the experimental protocol, conducted the study, and elaborated data. AS, SGi, AD, SGa, RG, CL, PL, and FF enrolled patients and organized experimental sessions. PM provided support for the organization and realization of the study. AS and FB edited the manuscript. FB supervised the entire experiment. All authors read and approved the final version of the article.

## Conflict of Interest

GC, AG, BI, and FB were employed by BrainSigns Srl. PM was employed by Cochlear Italia Srl. The remaining authors declare that the research was conducted in the absence of any commercial or financial relationships that could be construed as a potential conflict of interest.
